# Distribution of postpartum blood loss: modeling, estimation and application to clinical trials

**DOI:** 10.1186/s12978-018-0641-1

**Published:** 2018-12-04

**Authors:** José Ferreira de Carvalho, Gilda Piaggio, Daniel Wojdyla, Mariana Widmer, A. Metin Gülmezoglu

**Affiliations:** 1 Statistika Consultoria, Campinas, São Paulo Brazil; 2 Duke Clinical Research Institute, Duke University, Durham, North Carolina USA; 3 Department of Reproductive Health and Research, World Health Organization, UNDP/UNFPA/ UNICEF/WHO/World Bank Special programme of research, development and research training in human reproduction (HRP), Geneva, Switzerland

**Keywords:** Postpartum blood loss, Lognormal, Blood loss distribution, Postpartum haemorrhage, Clinical trials, Digit preference, Limit of detection of blood loss measures, Pérdida de sangre postparto, Lognormal, Distribución de pérdida de sangre, Hemorragia postparto, Ensayos clínicos, Preferencia de dígito, Límite mínimo de detección en medidas de pérdida de sangre

## Abstract

**Background:**

The loss of large amounts of blood postpartum can lead to severe maternal morbidity and mortality. Understanding the nature of postpartum blood loss distribution is critical for the development of efficient analysis techniques when comparing treatments to prevent this event. When blood loss is measured, resulting in a continuous volume measure, often this variable is categorized in classes, and reduced to an indicator of volume greater than a cutoff point. This reduction of volume to classes entails a substantial loss of information. As a consequence, very large trials are needed to assess clinically important differences between treatments to prevent postpartum haemorrhage.

**Methods:**

The authors explore the nature of postpartum blood loss distribution, assuming that the physical properties of blood loss lead to a lognormal distribution. Data from four clinical trials and one observational study are used to confirm this empirically. Estimates of probabilities of postpartum haemorrhage events ‘blood loss greater than a cutoff point’ and relative risks are obtained from the fitted lognormal distributions. Confidence intervals for relative risk are obtained by bootstrap techniques.

**Results:**

A variant of the lognormal distribution, the three-parameter lognormal distribution, showed an excellent fit to postpartum blood loss data of the four trials and the observational study. A measurement quality assessment showed that problems of digit preference and lower limit of detection were well handled by the lognormal fit. The analysis of postpartum haemorrhage events based on a lognormal distribution improved the efficiency of the estimates. Sample size calculation for a hypothetical future trial showed that the application of this procedure permits a reduction of sample size for treatment comparison.

**Conclusion:**

A variant of the lognormal distribution fitted very well postpartum blood loss data from different geographical areas, suggesting that the lognormal distribution might fit postpartum blood loss universally. An approach of analysis of postpartum haemorrhage events based on the lognormal distribution improves efficiency of estimates of probabilities and relative risk, and permits a reduction of sample size for treatment comparison.

**Trial registration:**

This paper reports secondary analyses for trials registered at Australian New Zealand Clinical Trials Registry (ACTRN 12608000434392 and ACTRN12614000870651); and at clinicaltrials.gov (NCT00781066).

**Electronic supplementary material:**

The online version of this article (10.1186/s12978-018-0641-1) contains supplementary material, which is available to authorized users.

## Abstracto

**Antecedentes:**

La pérdida de grandes cantidades de sangre después del parto puede llevar a morbilidad y mortalidad materna severas. Entender la naturaleza de la pérdida de sangre postparto es crítico para el desarrollo de técnicas de análisis eficientes cuando se comparan tratamientos para prevenir este evento. Cuando se mide la pérdida de sangre, que resulta en una variable continua, frecuentemente esta variable se categoriza en clases y se reduce a un indicador de volumen mayor que un cierto punto de corte. Esta reducción del volumen a categorías conlleva una pérdida sustancial de información. En consecuencia, son necesarios ensayos muy grandes para evaluar diferencias clínicamente importantes entre tratamientos de prevención de hemorragia postparto.

**Métodos:**

Los autores examinan la naturaleza de la distribución de pérdida de sangre postparto, suponiendo que las propiedades físicas de la pérdida de sangre resultan en una distribución lognormal. Los datos de cuatro ensayos clínicos y de un estudio observacional se usan como confirmación empírica. Las estimaciones de la probabilidad del evento hemorragia postparto ‘pérdida de sangre mayor que un cierto punto de corte’ y riesgos relativos se obtienen del ajuste de las distribuciones lognormales. Los intervalos de confianza para el riesgo relativo se obtienen usando técnicas de bootstrap.

**Resultados:**

Una variante de la distribución lognormal, la distribución lognormal con tres parámetros, produjo un excelente ajuste a los datos de pérdida de sangre postparto para los cuatro ensayos y para el estudio observacional. Una evaluación de la calidad de las medidas mostró que problemas de preferencia de dígito y de límite mínimo de detección eran bien controlados por el ajuste lognormal. El análisis del evento hemorragia postparto basado en la distribución lognormal mejoró la eficiencia de las estimaciones. El cálculo de tamaño de muestra para un futuro ensayo hipotético mostró que la aplicación de este procedimiento permite reducir el tamaño de muestra requerido para comparar tratamientos.

**Conclusión:**

Una variante de la distribución lognormal ajustó muy bien a los datos de pérdida de sangre postparto de diferentes áreas geográficas, sugiriendo que la distribución lognormal podría ajustar a la pérdida de sangre postparto universalmente. Una estrategia de análisis para los eventos de hemorragia postparto basada en la distribución lognormal mejora la eficiencia de las estimaciones de probabilidades y de riesgo relativo, y permite una reducción del tamaño de muestra para comparar tratamientos.

**Registro de los ensayos:**

Este artículo reporta análisis secundarios para ensayos registrados en los siguientes registros: Australian New Zealand Clinical Trials Registry (ACTRN 12608000434392 y ACTRN12614000870651); clinicaltrials.gov (NCT00781066).

## Plain English summary

The loss of large amounts of blood postpartum can lead to severe maternal morbidity and mortality. Understanding the nature of postpartum blood loss distribution is critical for the development of efficient analysis techniques when comparing treatments to prevent this event. When blood loss is measured, resulting in a continuous volume measure, often this variable is categorized in classes, and reduced to an indicator variable of blood loss greater than a certain cutoff point. This reduction of volume to an indicator variable entails a substantial loss of information. As a consequence, very large trials are needed to assess clinically important differences between treatments to prevent postpartum haemorrhage. Using data from four clinical trials and one observational study, the authors explore the nature of blood loss distribution and show that a variant of the lognormal distribution fits postpartum blood loss data from different geographical areas and times, thus suggesting that the lognormal distribution might fit postpartum blood loss universally. Based on this finding, they propose a lognormal approach of analysis of postpartum haemorrhage events of the type ‘blood loss greater than a certain cutoff point’, based on fitting a lognormal distribution to the data. The proposed approach improves efficiency and permits a reduction of sample size for treatment comparison.

## Background

Postpartum haemorrhage is a significant contributor to severe maternal morbidity, and the leading direct cause of maternal mortality worldwide [1–4]. Occurrence of postpartum haemorrhage (PPH) is defined as blood loss of 500 ml or more within 24 h after birth, and severe PPH (sPPH) as blood loss of 1000 ml or more [5].

It is now accepted that clinical trials conducted to compare treatments to prevent post partum haemorrhage should measure blood loss weight or volume, as opposed to subjective evaluation. It has been shown that the visual estimation underestimates the blood loss and that this underestimation increases when the loss is greater than 300 ml [6].

The estimation of PPH and sPPH is typically done by computing the sample proportions of women with measured blood loss above the pre-specified cutoff point corresponding to each of these events. Transforming a continuous variable to a dichotomous variable by grouping values into two or more categories may result in a considerable loss of power [7]. For a response variable, in a Monte Carlo study to investigate the effects of categorization of dependent variables on power to detect true effects, it was found that “the loss of power and required sample size increase were substantial under conditions in which the coarsely categorized variable is highly skewed, has few categories (e.g., 2, 3), or both.” [8] This is in fact the case of postpartum blood loss volume, which has a right-skewed distribution and is categorized in two categories. In trials to compare treatments to prevent postpartum haemorrhage, the use of an indicator variable, added to the low prevalence, results in very large sample sizes needed to detect improvements in prevention of blood loss endpoints when new treatments or procedures are evaluated.

Other caveats of categorization are the assumptions that there is a discontinuity in response as the cutoff point is crossed, and that the risk of maternal severe morbidity and mortality is the same for blood loss of, for example, 1001 mL and 1800 mL.

The development of a statistical analysis technique to analyze continuous postpartum blood loss volume depends on the knowledge of its distribution. The aim of this paper is to show empirically that the distribution of postpartum blood loss volume is lognormal, that the lognormal distribution can be used as a model for a lognormal analysis of postpartum blood loss, and to present the following applications of this finding for clinical trials: 1) Analyses using continuous blood loss volume based on the lognormal distribution improve the efficiency of comparisons of the proportions of sPPH and PPH between treatment groups. 2) As a consequence, the sample size needed to assess the difference of these proportions between treatments could be substantially reduced. 3) Researchers can also use outcomes based directly on the lognormal distribution parameters for many research questions, for example, to compare median blood loss values between two treatments or the proportions of women with blood loss greater than any cutoff point.

## The lognormal distribution as a model for postpartum blood loss

### The lognormal distribution and its applications

The lognormal distribution is a positively skewed distribution. If a variate has the lognormal distribution, transformation to logarithms results in a normal distribution.

The lognormal distribution has been used to model many problems in physics, chemistry, biology, geology and medicine [9]. In biology, the bacterial counts follow lognormal distributions [10]. The flow of rivers has also been shown to be lognormally distributed [11]. In medicine, examples include blood pressure [12], the dose of a drug required to cause a definite effect [13], the effect of drugs on enzymes and of oxygen on haemoglobin [13], measurements associated with the natural history of cancer, including survival and tumour size [14], and the volume of sweat [15].

The selection of a particular distribution to describe data is often based on both empirical evidence and on the physical and biological properties underlying the phenomenon that generates the variable to be analyzed. In the case of blood pressure, for example, empirical evidence suggested that the blood pressure age-specific distribution followed a lognormal distribution, and Makuch and Freeman developed the justification for the lognormal distribution as a model for blood pressure [12]. Fitting distributions to data to adequately represent a particular sample distribution without any other consideration than the closeness of the fitted to the empirical distribution might be useful in circumstances where substantive field properties are not known.

In the case of postpartum blood loss, there could be a physical reason to model the blood loss volume distribution by a lognormal distribution, namely that the increment in the volume of blood lost at a certain time could be assumed to depend on both the previous value of the volume and a random proportionate error. However, we will restrict our aim to show the quality of fit of the lognormal distribution to the data. For a derivation of the lognormal distribution from first principles, see, e.g., Johnson, Kotz, Balakrishnaia [9], and Aitchison and Brown [16].

If the postpartum blood loss volume (denoted as V) has the lognormal distribution, its probability density function (which gives the probability that V assumes a particular value v) is the following:

1 $$ f\left(v;m,s\right)=\frac{1}{v}\frac{1}{s\sqrt{2\pi }}\mathit{\exp}\left[\frac{-1}{2}{\left(\frac{\mathit{\log}(v)-m}{s}\right)}^2\right]\ \mathrm{for}\ \mathrm{any}-\infty <m<\infty, s>0\ \mathrm{and}\ \upsilon >0. $$

The parameters *m* and *s* are the mean and the standard deviation of *U = log(V)* respectively, which is a normal random variable. The parameter *exp(m)* is the median of the lognormal distribution. In what follows, *m* and *s* will be called location and scale parameters, respectively.

### Empirical evidence: Initial lognormal fits

Three large clinical trials have been conducted by WHO comparing two drugs [17, 18] or two management procedures [19] of the third stage of labour to prevent postpartum haemorrhage. Another (smaller) trial has been conducted by Althabe et al. comparing two management procedures [20]. The data for these trials were made available by the authors. We also present results from an observational study [21], based on published summarized results.

The characteristics of these studies are shown in (Additional file 1: Table A1). The four trials will be referred to as Misoprostol trial [17], Active Management trial [19], Althabe et al. trial [20], and CHAMPION trial [18]. The observational study will be referred to as Bamberg et al. study [21].

The methods to measure blood loss were similar but differed slightly across the studies: in the Misoprostol trial [17], blood loss was measured by collecting the blood in a jar to measure the volume. In the Active Management trial [19], the Althabe et al. trial [20] and the CHAMPION trial [18], the blood loss was measured by collecting the blood in a drape and weighing the drape. In the Bamberg et al. study [21], the blood was collected in a calibrated transparent plastic drape.

**Fig. 1:**
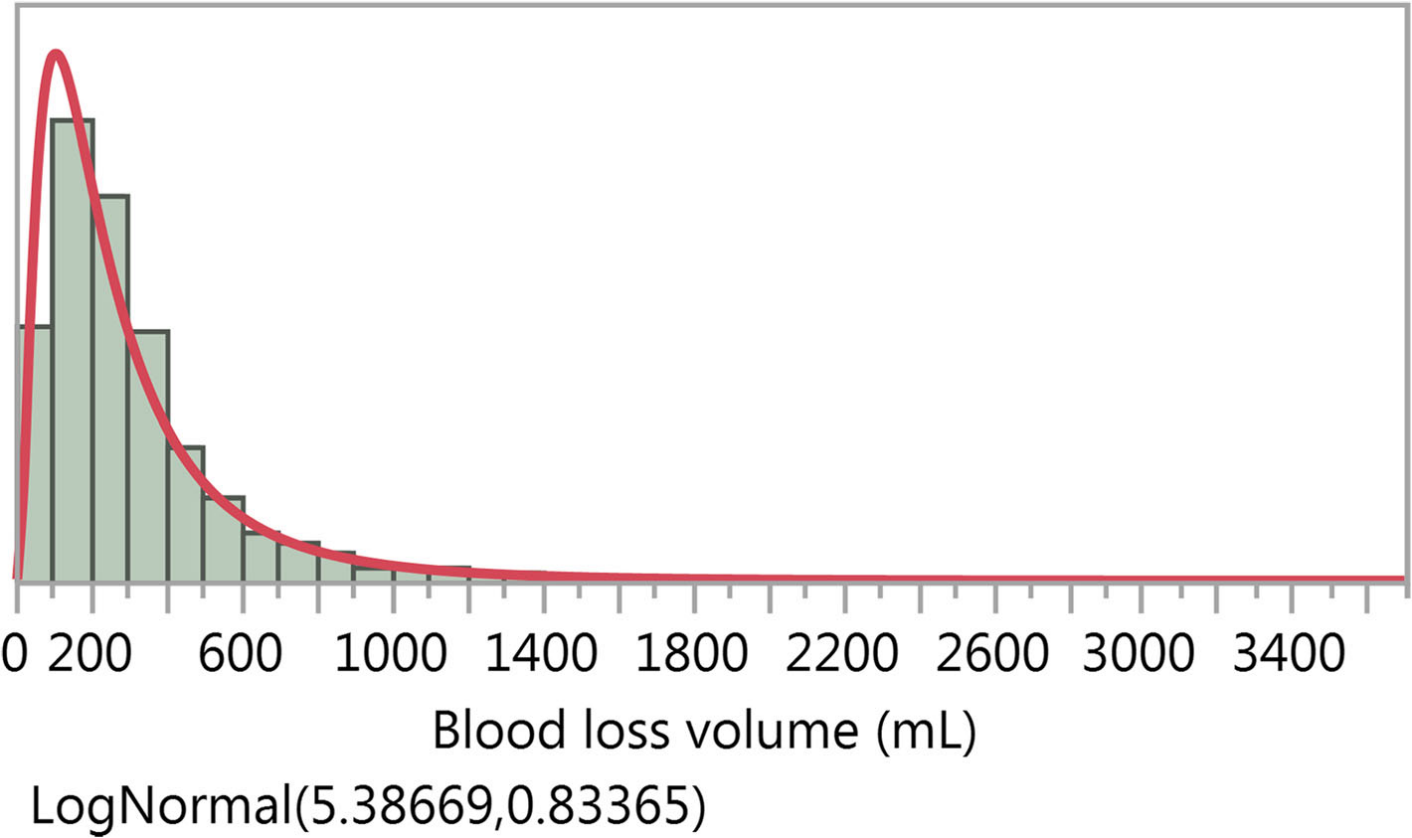
Blood loss volumes (mL) for the Simplified Package of the Active Management trial

We first described the blood loss data by standard histograms, probability plots and quantile-quantile plots (see explanation of probability and quantile-quantile plots in Appendix) from fitting the lognormal distribution (1) to data from the four trials listed above. We did this by treatment group for three of the trials [17, 19, 20], and for aggregated treatment groups for the CHAMPION trial, as the blood loss volume distributions were practically identical [18]. For illustration, Fig. 1 displays the histogram of the blood loss volumes for one treatment group of the Active Management trial, with the fitted lognormal density function superimposed (red curve). For all the trials the lognormal distribution showed a similar picture and one would visually conclude that it fitted to the data very well.

**Fig. 2:**
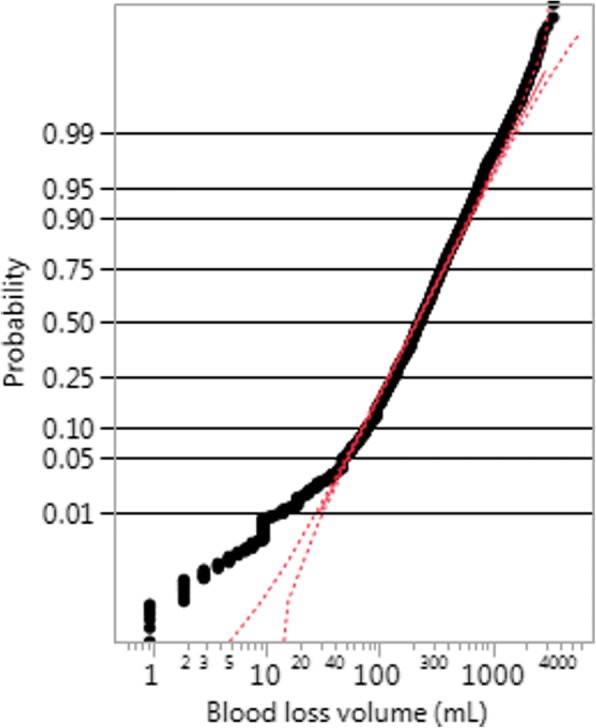
Probability plot for the fit of a two-parameter lognormal distribution for the Simplified Package of the Active Management trial

A closer look to goodness of fit, however, using probability plots, suggested that the fit was not very good for values of the volume V smaller than 50 mL. This is illustrated for the Active Management trial in Fig. 2, where the probability plot shows that the simple lognormal distribution is adequate to fit the blood loss volume distribution for volumes greater than 50 mL, but volumes below 50 mL cannot be measured with precision. We show this as an illustration, as the fits for all the four trials presented a very similar behaviour.

**Fig. 3:**
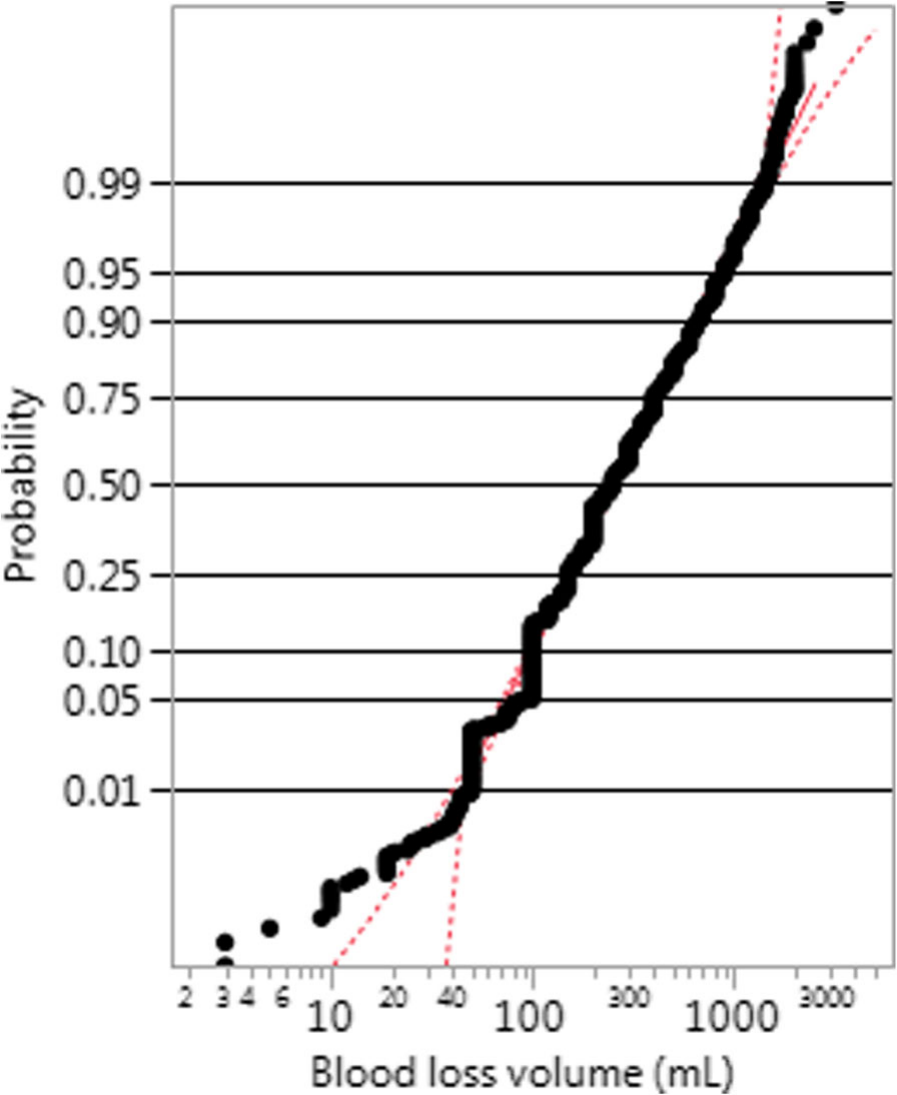
Probability plot for the fit of a two-parameter lognormal distribution for the Misoprostol treatment of the Misoprostol trial

When we look at the probability plot for the Misoprostol trial, shown in Fig. 3, we observe the same behaviour, namely that 1) for volumes above 100 mL the graph is fairly linear and within the confidence interval of the probability plot, even on the far right tail; 2) the fit is not good for volumes under 40, as the data points fall entirely off the confidence interval; but we see another interesting feature, that 3) the graph has a ragged aspect.

We decided then to have a closer look at measurements, which we describe in the next section.

### Measurement validation

#### Digit preference

It was noted that the measurements for the Misoprostol and Active Management trials clumped on multiples of 10, 50 and 100 mL and grams respectively. The same feature was not found for the CHAMPION trial. Table 1 shows the relative frequencies of values multiple of 10, 50 and 100 (mL or grams depending on the trial). In the table the observed relative frequencies can be compared with those expected if no clumping effect (digit preference) had been present.

**Table 1:** Expected and observed frequencies of data multiple of 10, 50 and 100 in the three large trials: Misoprostol trial [17], Active Management trial [19], CHAMPION trial [18]

Multiple of^a^	Expected frequency	Observed frequency		
		Misoprostol trial	Active Management trial	CHAMPION trial
10	0.10	0.90	0.35	0.09
50	0.02	0.59	0.09	0.02
100	0.01	0.42	0.03	0.01

^a^The unit is mL in the Misoprostol trial and grams in the Active Management and CHAMPION trials

**Fig. 4:**
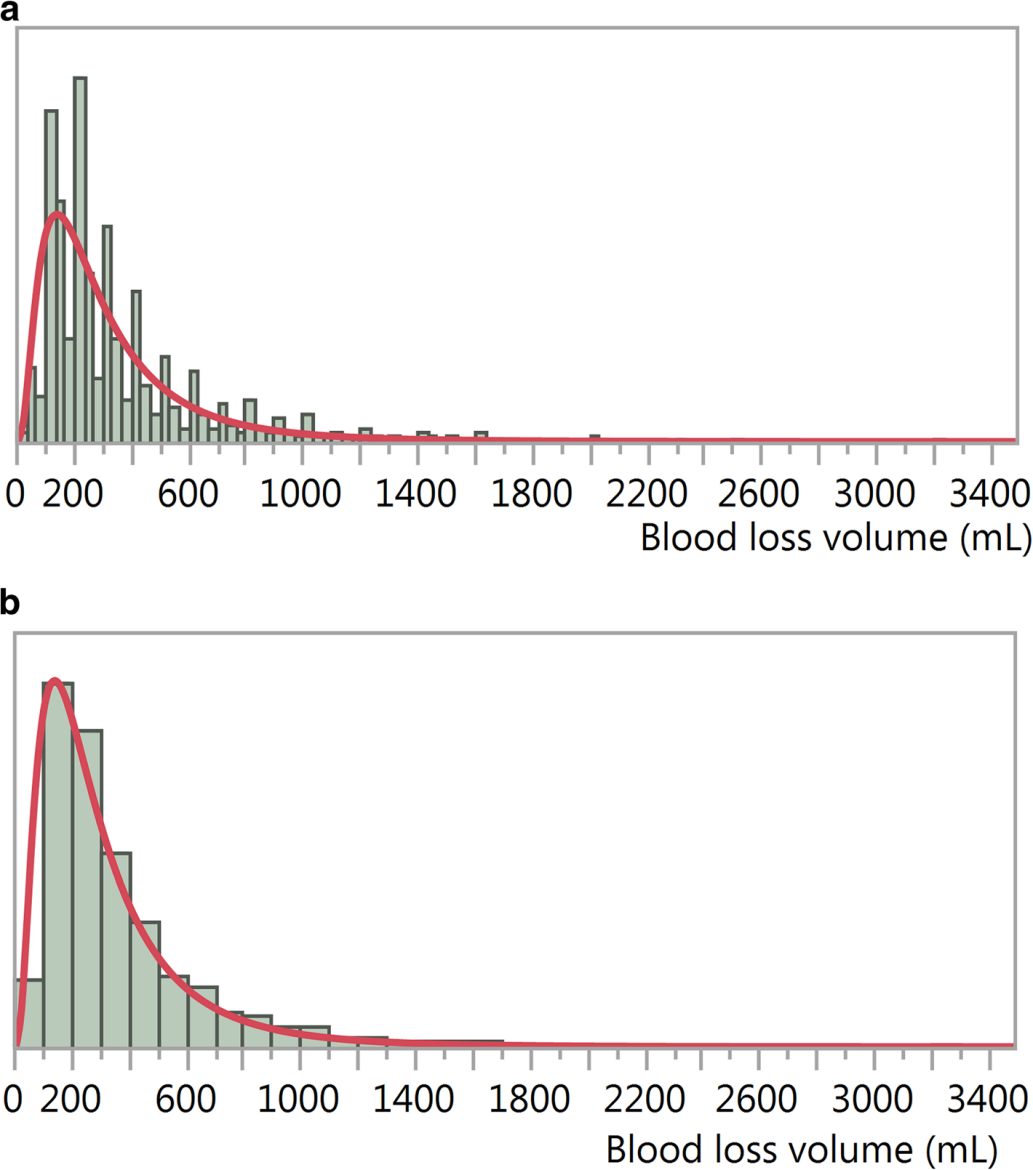
Histograms **a**: showing digit preference (narrow bins) and lognormal fit (red line), and **b**: masking digit preference (bins of 100 mL) and lognormal fit (red line), Misoprostol treatment, Misoprostol trial

Table 1 shows that the values recorded for the Misoprostol trial have been rounded to multiples of 100 more than expected, and this explains the ragged aspect of the line in Fig. 3. The same effect happened with the Active Management trial, but to a lesser degree. There is no evidence of this rounding for the CHAMPION trial data. The digit preference feature is illustrated for the Misoprostol trial using histograms (Fig. 4).

The digit preference feature might have an impact on the binomial estimates of the proportions of PPH and sPPH, introducing a positive bias when values are rounded to nearest 100. As it is expected that the frequencies on the right tail of the distribution will decrease monotonically with the volume, the amount moved from the left neighboring class will appear in excess of the corresponding points on the right neighboring class, therefore increasing the estimate of the event “greater than 1000 (say)”. The difference in shifted points proportions might be large compared to the proportion of sPPH.

The possible bias arising from rounding might also result in an overestimation of the variance of the estimator, as the measurement error is also subject to variation.

#### Limit of detection

Every system of measurement has a limit of detection (LOD). Values below the LOD will not be measured precisely [22].

As shown previously in Fig. 2, measurements below 50 mL seem to be measured with low precision, suggesting that the LOD for this measurement is around 50 mL. This finding was also implicit in Bamberg et al’s paper, which reports volumes in multiples of 50 mL [21].

Measurements below 50 mL were distributed as a uniform variate for all trials. For the Misoprostol trial, in particular, values below 100 mL were distributed as a uniform random variable. This shows that the volumes registered below 50 mL (Active Management trial) or below 100 mL (Misoprostol trial) are random values between 0 and 50 or between 0 and 100 respectively.

Note however that this imprecision of measurements in the left tail of the distribution will not affect direct estimates of proportions of sPPH and PPH, which involve the right tail. It is, however, affecting the fit of a simple lognormal distribution, and we will describe how we corrected for this in the next section.

### Correcting for measurement problems: The three-parameter lognormal distribution

We corrected the deviation from the lognormal distribution due to values reported below the LOD, using the three-parameter lognormal distribution (see, e.g. Meeker and Escobar 1998, page 111 ff) [23], that has the following probability density function:

2 $$ {f}_V\left(v;m,s,t\right)=f\left(v-t;m,s\right)\mathrm{for}\ v>t, $$

where f is the lognormal density defined in (1).

The third parameter t improved the goodness of fit.

### Empirical evidence: Final lognormal fits

Table 2 presents the estimated parameters in each trial, by treatment or intervention (Misoprostol, Active Management, Althabe et al. trials) or aggregated (CHAMPION trial).

**Table 2:** Estimated parameters for the fitted three-parameter lognormal distribution for the five studies, with two-sided 95% confidence intervals (CI) (the location parameter is *m* in formula (3), the scale parameter is *s*, and the third parameter is t): Misoprostol trial [17], Active Management trial [19], CHAMPION trial [18], Althabe et al. trial [20] and Bamberg et al. study [21]

Trial	Treatment	Parameter	Estimate	Std Error	95% CI	
					Lower limit	Upper limit
Misoprostol	Misoprostol	location	5.58	0.011	5.556	5.600
		scale	0.71	0.008	0.695	0.726
		t	−8.60	1.705	−11.945	−5.261
	Oxytocin	location	5.46	0.011	5.437	5.482
		scale	0.69	0.008	0.677	0.708
		t	−12.01	1.654	−15.249	−8.765
Active Management	Simplified Package	location	5.63	0.013	5.609	5.658
		scale	0.63	0.008	0.611	0.643
		t	−47.38	2.549	−52.373	−42.382
	Full Package	location	5.57	0.012	5.550	5.598
		scale	0.65	0.008	0.632	0.664
		t	−43.53	2.300	−48.034	−39.019
CHAMPION	Aggregated	location	5.19	0.009	5.167	5.204
		scale	0.83	0.007	0.812	0.840
		t	−22.25	0.937	−24.089	− 20.415
Althabe et al	Hands Off	location	5.57	0.141	5.299	5.850
		scale	0.72	0.101	0.522	0.917
		t	55.14	24.474	7.176	103.113
	CCT	location	5.37	0.132	5.110	5.628
		scale	0.80	0.101	0.600	0.997
		t	62.88	16.414	30.709	95.049
Bamberg et al	–	location	5.47	–	–	–
		scale	0.66	–	–	–

**Fig. 5:**
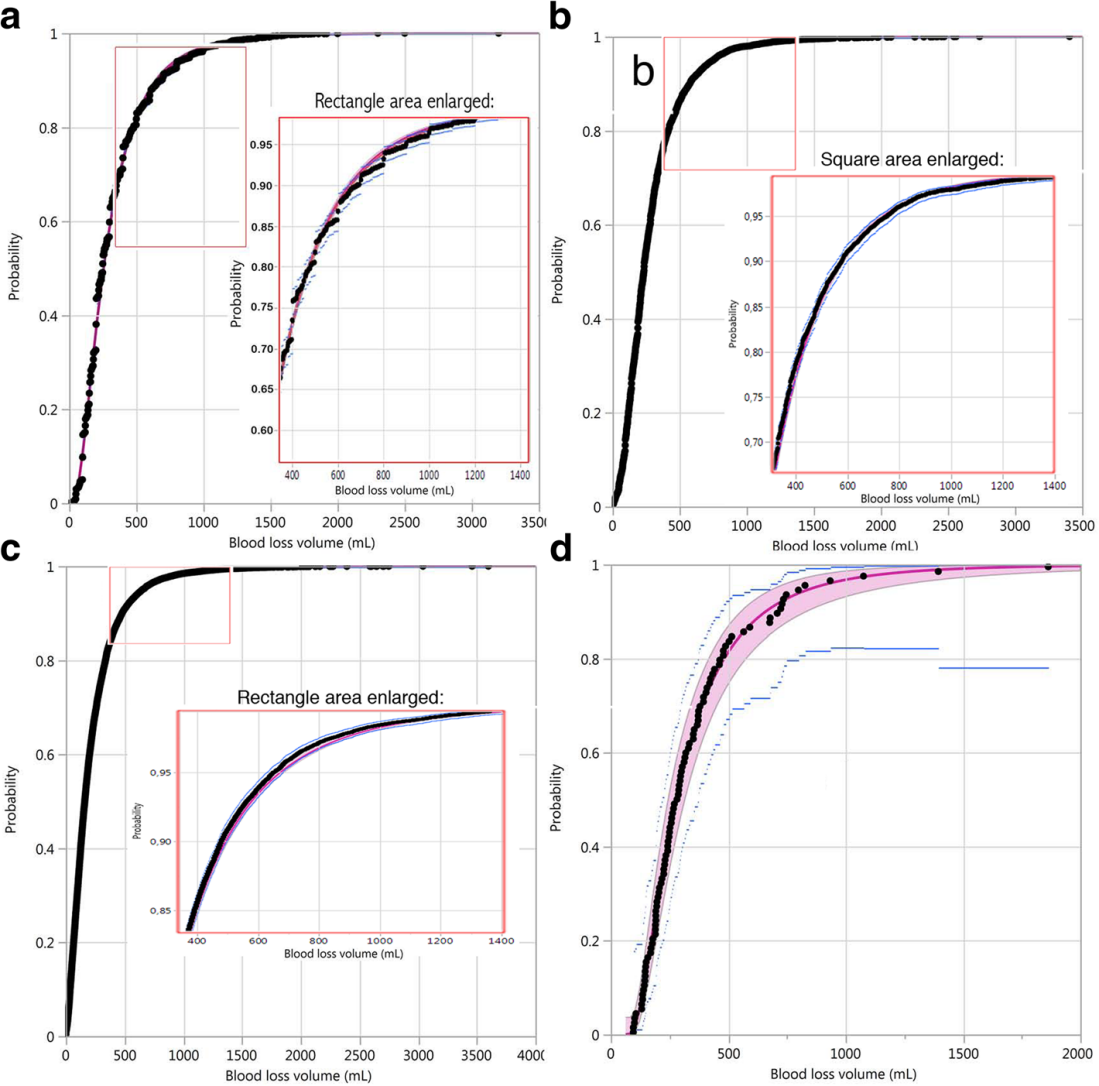
Empirical cumulative distribution function (black dots) and three-parameter lognormal fit (red line); **a**: Misoprostol treatment, Misoprostol trial; **b**: Simplified package, Active Management trial; **c**: aggregated treatments, CHAMPION trial; **d**: CCT treatment, Althabe et al. trial, showing also 95% CIs for the fitted lognormal cumulative distribution (red area) and for the empirical cumulative distribution (blue lines); a magnified area is shown for **a**, **b** and **c** in the range of 400 to 1400 mL; only one treatment shown for the first two and the last trial, as the graphs for the two treatments were almost identical

**Fig. 6:**
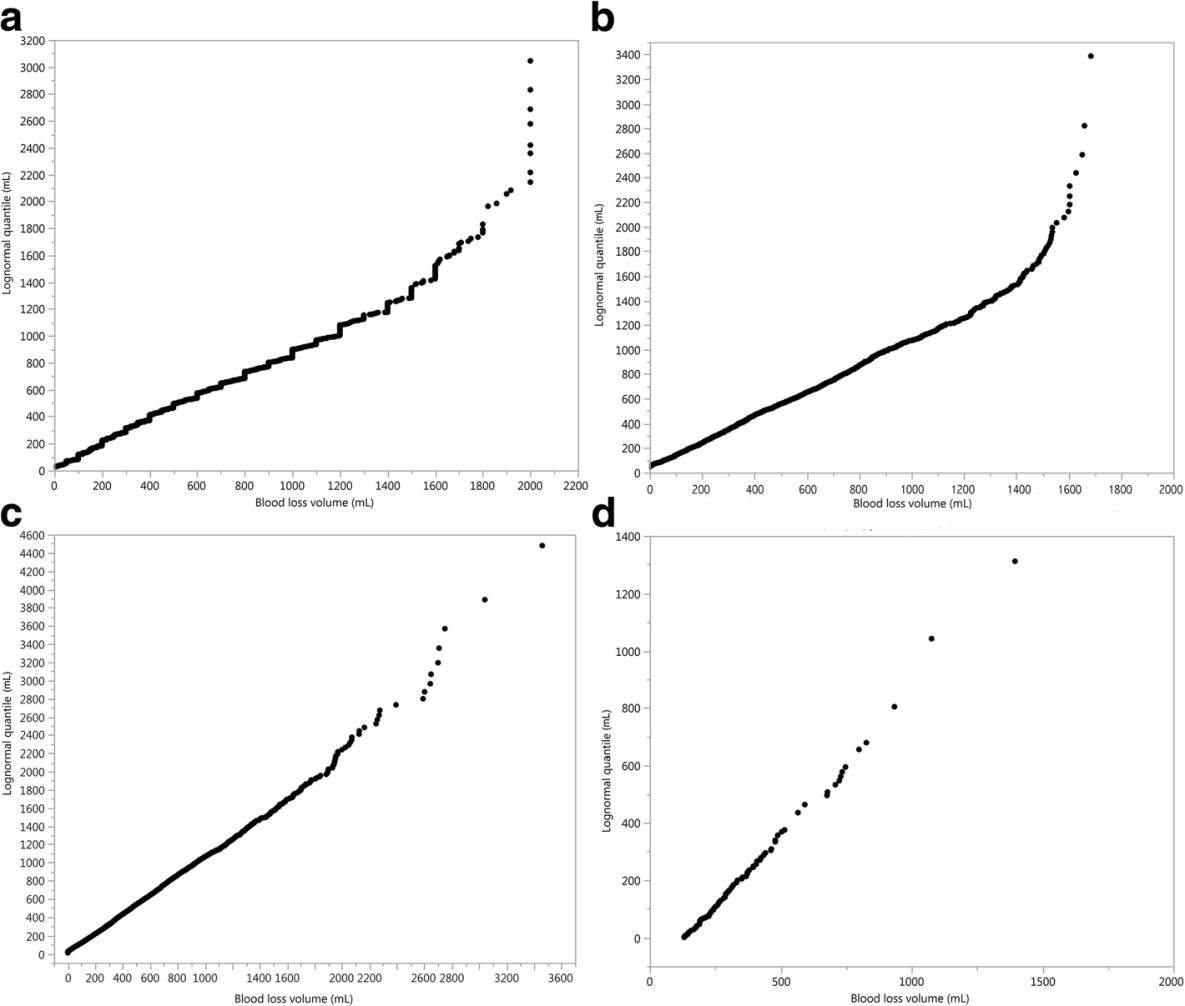
Quantile-quantile plot of the fitted three-parameter lognormal distribution quantiles versus observed blood loss volume quantiles (mL), showing a good fit up to 1800 mL (Misoprostol treatment, Misoprostol trial, panel **a**), up to 1500 mL (Active Management trial, panel **b**), and in all the range (CHAMPION trial, panel **c**, and Althabe et al. trial, panel **d**); only one treatment shown for the first two and the last trial, as the graphs for the two treatments were almost identical

Figures 5 and 6 provide evidence for the suitability of the three-parameter lognormal distribution as a good model for postpartum blood loss volumes. Figure 5 shows the observed values in overlay with the fitted lognormal cumulative distribution functions for each of the four trials. For the three large trials (panels a, b and c) the observed and fitted values and confidence intervals were so close that they were completely confounded in the graph when including the whole range. Therefore we have inserted a magnified area in the range 400 to 1400 mL, showing better the four elements of the graph, which are still clearer and more distinct for the smaller trial (panel d): observed values (black dots) well in agreement with the fitted lognormal curve (red line); 95% confidence band for the lognormal estimates (red area), narrower than the 95% confidence interval for the binomial estimates (blue lines). From these graphs we can conclude: 1) there is an excellent fit of the three-parameter lognormal distribution to the blood loss data of the four trials; 2) the precision of estimates of the proportion of events of the type blood loss greater than a certain value is greater with the lognormal approach compared to the binomial approach.

Figure 6 presents quantile-quantile plots, where the straight line demonstrates a very good fit up to 1800 mL (Misoprostol trial, panel a), 1500 mL (Active Management trial, panel b) and in all the range (CHAMPION and Althabe et al. trials, panels c and d respectively).

Goodness of fit statistics (Akaike Information Criterion and Bayes Information Criterion) for the three-parameter lognormal and other candidate distributions (lognormal, Smallest Extreme Value, Largest Extreme Value, Log Generalized Gamma) also show that this variant of the lognormal distribution fits the data very well (see Additional file 2). Quantiles for the fitted three-parameter lognormal distribution and for the empirical distributions for the four trials, corresponding to Fig. 5, have been calculated (see Additional file 3).

Table 2 also shows the results for the Bamberg et al. study [21], which reported five quantiles (0.05, 0.25, 0.50. 0.75 and 0.95) of postpartum blood loss for 739 deliveries. Using the reported quantiles we recovered the distribution by finding, within the lognormal family of distributions, the one with quantiles closest to the series of quantiles presented, applying the solution implemented by Belgorodski et al. [24] in R software (package rriskDistributions).

**Fig. 7:**
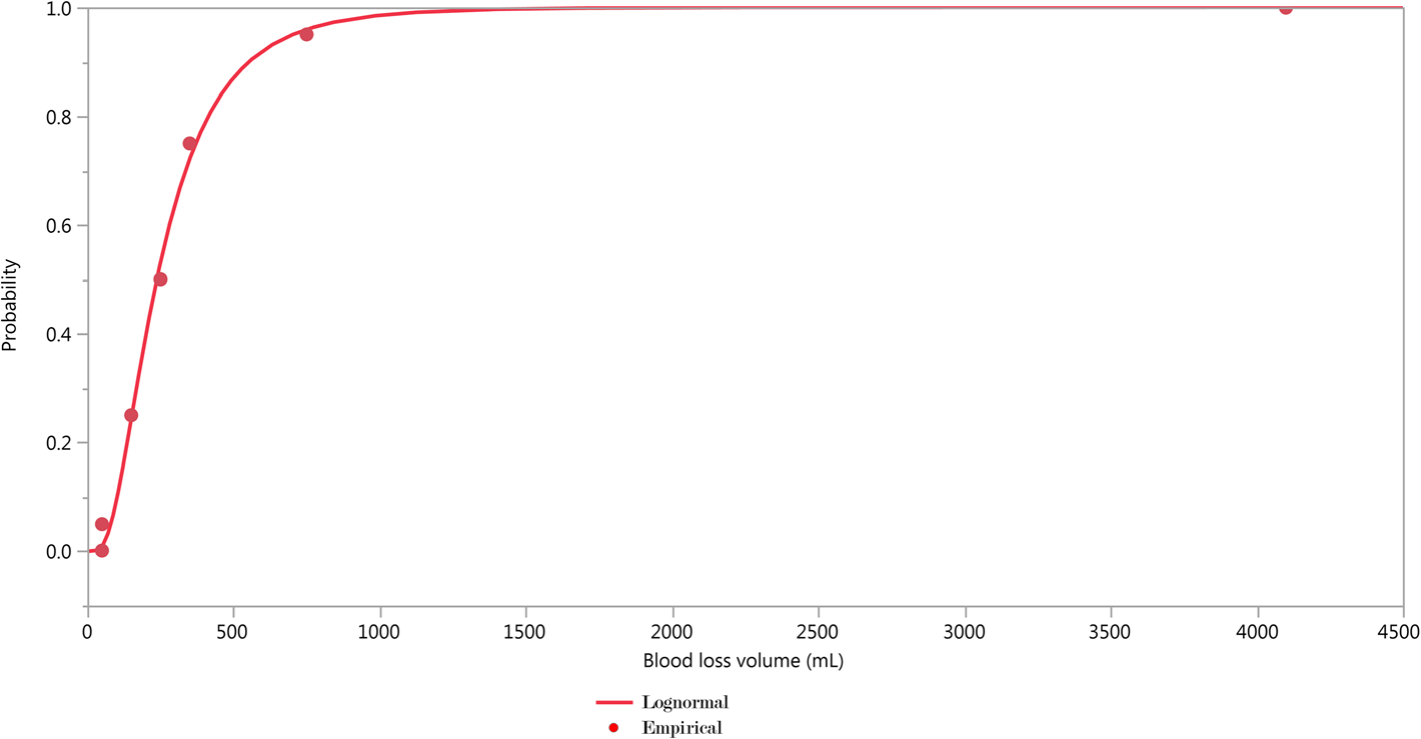
Quantiles reported in Bamberg et al. observational study (dots) and fitted lognormal distribution (full line)

Figure 7 shows the results of fitting the lognormal distribution using the quantiles reported in Bamberg et al. paper [21]. In the last two lines of Table 2 we present the estimates of the parameters of the lognormal distribution, m = 5.47 and s = 0.66, well in line with the results of the four trials.

The estimated location and scale parameters are very similar across the different studies. The trials were done in different places and times, so it seems that the lognormal distribution fits postpartum blood loss data universally. The third parameter varies more widely, probably due to the blood loss measurement procedure rather than to the true blood loss volume.

## Applications

### Improving the precision of estimates

#### Estimating the proportions of sPPH and PPH

The estimated parameters of the fitted three-parameter lognormal distribution shown in Table 2 were estimated by maximum likelihood, for each of the five studies. The estimates of sPPH and of PPH are simply the values of the complement of the fitted cumulative distribution function (the probability of larger value) at the points 1000 and 500, respectively. These are computed directly from the lognormal estimated parameters, which are estimated by maximum likelihood, and therefore they are also maximum likelihood estimators. All the asymptotic distributional properties of the maximum likelihood estimators apply, and therefore confidence intervals and hypothesis tests are readily available. This estimation approach will be denoted the lognormal approach.

On the other hand, the approach of computing the sample proportion of women with blood loss above a cut off point, or binomial proportion, will be denoted the binomial approach. We provide the maximum likelihood based confidence limits for this binomial proportion.

In Tables 3 and 4 we show estimates of sPPH and PPH respectively, obtained by the binomial approach and by the lognormal approach. As shown in Table 3, the differences between the binomial and the fitted three-parameter lognormal estimates of sPPH are of the order of 0.3% on the absolute scale for the Active Management and the CHAMPION trials, and the 95% confidence intervals overlap. For the Althabe et al. trial the 95% confidence intervals also overlap, and they are wider as this was a small trial. For the Misoprostol trial the 95% confidence intervals do not overlap. We attribute this to the imprecision resulting from rounding (see section on Digit preference), that affects more drastically the binomial estimate.

**Table 3:** Estimated proportions of sPPH for the four trials, with two-sided 95% confidence intervals (CI), by the binomial and by the lognormal approaches: Misoprostol trial [17], Active Management trial [19], CHAMPION trial [18] and Althabe et al. trial [20]

Trial	Treatment	Proportion	95% CI		Width of the 95%CI	Width ratio lognormal vs binomial (%)
			Lower limit	Upper limit		
Binomial						
Misoprostol	Misoprostol	0.03962	0.03582	0.04380	0.00797	–
	Oxytocin	0.02839	0.02520	0.03199	0.00679	–
Active Management	Simplified Package	0.02057	0.01814	0.02331	0.00517	–
	Full Package	0.01885	0.01653	0.02148	0.00495	–
CHAMPION	Aggregated	0.01560	0.01400	0.01739	0.00339	–
Althabe et al	Hands Off	0.05102	0.02199	0.11392	0.09194	–
	CCT	0.02970	0.01015	0.08372	0.07356	–
Lognormal						
Misoprostol	Misoprostol	0.02976	0.02740	0.03229	0.00490	61.4
	Oxytocin	0.01748	0.01582	0.01928	0.00346	51.0
Active Management	Simplified Package	0.01760	0.01603	0.01930	0.00327	63.2
	Full Package	0.01685	0.01532	0.01850	0.00317	64.0
CHAMPION	Aggregated	0.01737	0.01617	0.01865	0.00248	73.2
Althabe et al	Hands Off	0.03799	0.01655	0.07807	0.06151	66.9
	CCT	0.03250	0.01369	0.06885	0.05515	75.0

**Table 4:** Estimated proportions of PPH for the four trials, with two-sided 95% confidence intervals (CI), by the binomial and by the lognormal approaches: Misoprostol trial [17], Active Management trial [19], CHAMPION trial [18] and Althabe et al. trial [20]

Trial	Treatment	Proportion	95% CI		Width of the 95%CI	Width ratio lognormal vs binomial (%)
			Lower limit	Upper limit		
Binomial						
Misoprostol	Misoprostol	0.19462	0.18666	0.20283	0.01617	–
	Oxytocin	0.13526	0.12843	0.14239	0.01396	–
Active Management	Simplified Package	0.13751	0.13137	0.14389	0.01252	–
	Full Package	0.12847	0.12251	0.13468	0.01217	–
CHAMPION	Aggregated	0.09128	0.08743	0.09530	0.00787	–
Althabe et al	Hands Off	0.22449	0.15318	0.31658	0.16340	–
	CCT	0.16832	0.10783	0.25311	0.14528	–
Lognormal						
Misoprostol	Misoprostol	0.17866	0.17236	0.18510	0.01275	78.8
	Oxytocin	0.13030	0.12481	0.13596	0.01115	79.9
Active Management	Simplified Package	0.14198	0.13687	0.14722	0.01035	82.7
	Full Package	0.13192	0.12697	0.13701	0.01004	82.5
CHAMPION	Aggregated	0.09709	0.09381	0.10046	0.00665	84.5
Althabe et al	Hands Off	0.23344	0.17098	0.30684	0.13586	83.1
	CCT	0.18662	0.13103	0.25485	0.12382	85.2

In Table 4 we observe a similar picture for PPH, with confidence intervals overlapping except for one of the treatments of the Misoprostol trial.

The confidence intervals are shorter for the lognormal based estimates. For the sPPH proportion, the ratio of the width of the 95% confidence interval of the lognormal approach in relation to that of the binomial approach varies around 60% (from 51 to 75%). For the PPH proportion, the width ratios are around 80% (from 79 to 85%).

#### Estimating relative risks

We computed relative risks to compare the two treatments for the four trials, as in the published results [17–20]. We defined a relative risk (RR) as the ratio of two proportions, the proportion of women with sPPH (or PPH) in one arm to the proportion of women with sPPH (or PPH) in the other arm. For the binomial approach, confidence intervals for the RR were computed using maximum likelihood. For the lognormal approach, confidence intervals for the RR were computed using bootstrap. One thousand bootstrap samples were generated for each treatment. The estimates of the proportions of sPPH and PPH were computed for each bootstrap sample, for each treatment. Following Efron and Tibshirani [25], the two bootstrap samples tables were matched by row (sample) and the RRs computed. From the distribution of the 1000 bootstrapped RRs, the confidence interval was obtained.

The RRs with 95% confidence intervals for sPPH are shown in Table 5 for both the binomial and the lognormal approach. The confidence intervals are narrower for the lognormal approach than for the binomial approach, with the only apparent exception of the Misoprostol trial for the sPPH outcome (for which the widths of the confidence intervals were 0.44 for both approaches). However, we must take into account that the variances are estimated assuming exact observations, ignoring the fact that there is error in measurements. Other than this, the confidence intervals for the RR are shorter for the lognormal based estimates, varying from 57 to 73% for sPPH, and from 72 to 84% for PPH.

**Table 5:** Estimated relative risks of sPPH and PPH for four trials, with two-sided 95% confidence intervals (CI), by the binomial and by the lognormal approaches: Misoprostol trial [17], Active Management trial [19], CHAMPION trial [18] and Althabe et al. trial [20]

Event Approach	Trial	RR	95% CI		Width of the 95% CI	Width ratio lognormal vs binomial (%)
			Lower limit	Upper limit		
sPPH						
Binomial	Misoprostol	1.39	1.19	1.63	0.44	–
	Active Management	1.09	0.91	1.31	0.40	–
	CHAMPION	1.04	0.87	1.25	0.38	–
	Althabe et al	1.76	0.40	7.56	7.16	–
Lognormal	Misoprostol	1.69	1.48	1.92	0.44	100.0
	Active Management	1.05	0.91	1.20	0.29	72.5
	CHAMPION	1.00	0.89	1.13	0.24	63.2
	Althabe et al	1.16	0.38	4.44	4.06	56.7
PPH						
Binomial	Misoprostol	1.44	1.35	1.54	0.19	–
	Active Management	1.07	1.00	1.14	0.14	–
	CHAMPION	0.99	0.92	1.06	0.14	–
	Althabe et al	1.33	0.76	2.35	1.59	–
Lognormal	Misoprostol	1.37	1.29	1.45	0.16	84.2
	Active Management	1.08	1.02	1.14	0.12	85.7
	CHAMPION	1.01	0.95	1.06	0.11	78.6
	Althabe et al	1.26	0.81	1.95	1.14	71.7

### Sample size

Consider the sample size determination for the comparison of two drugs to prevent sPPH. Let us suppose that one drug is in current use and the rate of sPPH with this drug is 2%. Assume that a change to the new drug would be worth if it results in a sPPH rate below 1.5% (a change of 0.75 on the relative scale, or an improvement of 25%). Further assume a 5% significance level and a power of 80% for this test. For the sake of simplicity, we assume a completely randomised experiment (also known as ‘parallel group design’), a superiority hypothesis and a one-sided test.

The sample size for the binomial-based statistic is computed by standard procedures, based on the asymptotic normal distribution of the difference estimator. The result (without correction for continuity) is 17,008 participants (8504 in each treatment group).

To determine sample size based on the lognormal distribution, we rely on the normal distribution, by transforming the requirements expressed in the volume V to equivalent requirements in log(V). For the standard deviation s, we use the results from the fittings of the lognormal distribution, s = 0.7, the estimated standard deviation of the distribution of log(V) (all the fits gave approximately this value for s).

The sPPH proportion is.

3 $$ p=P\left(V>1000|m,s=0.7\right) $$

and the two values to be compared are 0.02 and 0.015.

The expression (3) is equivalent to.

$$ P\left(\log (V)>\log (1000)|m,s=0.7\right) $$

where log(V) has the normal distribution. Therefore

$$ P\left(Z>\frac{\left[\mathit{\log}(1000)-\mathrm{m}\right]}{0.7}\right)=\mathrm{p} $$

where Z has the standard normal distribution, and comparing *p* = 0.02 vs *p* = 0.015 from the lognormal distribution is equivalent to comparing the two corresponding values of *m* from the normal distribution.

From the standard normal distribution, z_0.985_ = 2.1701 and z_0.98_ = 2.0537. The solutions for m are:

$$ \mathrm{for}\ \mathrm{p}=0.015,\mathrm{m}=\log (1000)-2.1701\ \mathrm{x}\ 0.7=5.3887; $$

$$ \mathrm{for}\ \mathrm{p}=0.02,\kern0.5em \mathrm{m}=\log (1000)-2.0537\ \mathrm{x}\ 0.7=5.4701. $$

It is sufficient to calculate the sample size to compare two means of normal distributions, with the requirements set forth above. The solution is a total sample size of 1832, to be divided equally in two groups of 916 units.

### Inference based on the lognormal parameters directly

When a variate measured in the original units can be described by a lognormal distribution, transformation to logarithms results in a normal distribution. Tests and confidence intervals can be computed on the basis of normal theory, and confidence limits can be re-transformed to the original scale.

Since the blood loss volume is lognormally distributed, several interesting consequences follow. The distribution is characterized by two parameters, *m* and *s* (and eventually, a third parameter *t*). The statistics given by the mean of the logarithms of the observed volumes,

$$ \widehat{m}=\frac{1}{n}\sum \limits_{i=1}^n\log \left({v}_i\right), $$

and the sample variance of the logarithm of V,

$$ {\widehat{s}}^2=\frac{1}{n}\sum \limits_{i=1}^n{\left(\log \left({v}_i\right)-\widehat{m}\right)}^2 $$

are jointly sufficient for the parameters. Hence, every inference can be based on these two statistics.

The most efficient estimators of *m* and *s* are given by the mean and the standard deviations of the logarithms of the volumes, and the usual properties of inference based on the normal distribution apply.

The outcomes used in clinical trials, the complement of the cumulative distribution function at 500 mL and 1000 mL, can be readily computed from the knowledge of these two statistics. There might be other hypotheses of interest, like the comparison of two medians.

Given that the variance is relatively stable (as shown in Table 2, the estimate of the parameter *s*, varied between 0.7 and 0.8 across the five studies), it seems reasonable to base comparisons on the medians [26]. For fixed *s*, in a comparison of two groups, *m*_*1*_ *< m*_*2*_ implies that the probability of a larger value with treatment 1 is smaller than with treatment 2. Then we can use common statistical knowledge, estimating and comparing medians, taking into account that the difference of means of log(V) is equivalent to the ratio of medians on the untransformed scale [26].

## Discussion

Several treatments for preventing postpartum haemorrhage have been compared in clinical trials, and other postpartum haemorrhage clinical trials are expected to be conducted in the future. Severe postpartum haemorrhage (sPPH) occurs at rates of 1 to 4% typically, varying according to time and geographical region [1]. When comparing treatments or interventions to prevent this event, the available technique for analysis up to date was to estimate the two binomial proportions of sPPH, demanding very large and costly clinical trials.

Using postpartum blood loss data available from four trials and quantiles from one observational study we have shown that the blood loss volume distribution can be very well represented by the three-parameter lognormal distribution. Using this finding, we showed that the precision of estimates of proportion of events of the type ‘blood loss more than a cut off point’ is improved, as well as the comparison of these proportions using relative risk.

When postpartum blood loss weight or volume is measured, we suggest to make use of the richness provided by this continuous variable in the analysis. The procedure we propose consists of the following steps: first fit a lognormal distribution to the measured blood loss data. The parameters of the lognormal distribution can be estimated by maximum likelihood. Once the cumulative distribution function of the volume or its complement is defined by its parameters, the proportion of sPPH, for example, is just the complement distribution at the point 1000. To compare treatments and compute relative risks with confidence intervals, we propose using bootstrap techniques. A step-by-step illustration of the proposed approach, using the data of the Althabe et al. trial [20], has been published [27].

Other variables in medicine and biology related to size, mass and volume have been conveniently represented by the lognormal distribution [12–14], and the procedure we propose could also be applied to these variables.

The most important and appealing application of using the lognormal model for the distribution of the blood loss volume is the possibility of a substantial reduction of sample size in clinical trials, with consequent reduced cost, while keeping the statistical precision requirements. This is achieved through an improvement in the efficiency of the estimation methods. In addition, more manageable sizes can ensure that data quality is better preserved.

A possible limitation of the approach we propose is whether the lognormal distribution is appropriate for modeling postpartum blood loss when there is a strong interest in the estimation of tail probabilities of the order of magnitude of 1 to 4%. However, the techniques that we used to assess the goodness of fit, such as cumulative distribution function, probability plots, and quantile-quantile plots, showed that the fit was satisfactory for volumes of more than 1000 mL. If interest arises for estimating more extreme tail probabilities (more than 2000 mL, say), special techniques should be considered [28]. Such a problem would be of interest for blood storage for transfusions, which is an even more rare event.

Another limitation is the additional statistical analysis work needed to fit the three-parameter lognormal distribution to the data and to compute confidence intervals for the relative risk using bootstrap. However, this can be done easily with current computer facilities available.

## Conclusions

Our results suggest that a variant of the lognormal distribution fits postpartum blood loss universally. Based on this finding, we propose a lognormal approach of analysis of postpartum haemorrhage trials aiming to compare events of the type ‘blood loss greater than a certain cutoff point’ across treatments, based on the lognormal distribution. As using all the measured data provides more information than a categorization [7, 8], we expected the estimators based on the lognormal approach to fare better than the binomial estimators, and we showed empirically that this is the case: the estimates based on the lognormal approach were more precise than those based on the binomial approach. The proposed approach improves efficiency and permits a reduction of sample size for treatment comparison.

## Additional files

Additional file 1: **Table A1.** Characteristics of studies analysed for blood loss: four randomised clinical trials (RCTs), one observational study. (PDF 92 kb)

Additional file 2: **Table A2.** Goodness of fit statistics for the three-parameter lognormal and other distributions, by treatment (a: Misoprostol trial; b: Active Management trial; d: Althabe et al. trial), or aggregated treatments (c: CHAMPION trial). (PDF 134 kb)

Additional file 3: **Table A3.** Quantiles for the fitted three-parameter lognormal and empirical distributions, with 95% CI, by treatment (a: Misoprostol trial; b: Active Management trial; d: Althabe et al. trial), or aggregated treatments (c: CHAMPION trial). (PDF 145 kb)

## Appendix: definitions

### Probability plot

The **probability plot** is a graphical technique for assessing whether or not a data set follows a given distribution such as the lognormal. The data are plotted against the lognormal distribution. If the distribution of the data is lognormal, the points will approximately lie on the line *y* = *x*.

### Quantile-quantile plot

A **quantile**-**quantile** (**q-q**) **plot** is a graphical technique for determining if two data sets come from populations with a common distribution. In our case we compare the distribution of fitted values with the empirical (observed) distribution, therefore the **q-q plot** is a **plot** of the **quantiles** of the fitted lognormal distribution against the **quantiles** of the observed values. If the two distributions being compared are similar, the points in the q–q plot will approximately lie on the line *y* = *x*.

## Abbreviations

HRP: UNDP/UNFPA/UNICEF/WHO/World Bank Special Programme of Research, Development and Research Training in Human Reproduction
LOD: Limit of detection
PPH: Postpartum haemorrhage
RR: Relative risk
sPPH: Severe postpartum haemorrhage
V: Blood loss volume
