# Supplementary material for: Distribution of postpartum blood loss: modeling, estimation and application to clinical trials
Source: Reprod Health. 2018 Dec 4;15:199. doi: 10.1186/s12978-018-0641-1 (PMC6280488; doi:10.1186/s12978-018-0641-1)
Supplement: Supplementary file 1 — Table A1. Characteristics of studies analysed for blood loss: four randomised clinical trials (RCTs), one observational study. (PDF 92 kb) [file 12978_2018_641_MOESM1_ESM.pdf]

Table A1. Characteristics of studies analysed for blood loss: four randomised clinical trials (RCTs), one observational study

	RCT or observational	Multicenter	Blind or Open-label	Type of hypotheses to compare treatments	Treatments	Outcome	Method to measure blood loss	Trial sample size*	Effective sample size*
Misoprostol trial (Gülmezoglu 2001[17])	RCT	Yes (9 countries worldwide)	Double-blind	Equivalence	Misoprostol, Oxytocin	sPPH primary, PPH secondary	Jar-Volume	18530	18442
Althabe et al trial (Althabe 2009[20])	RCT	Yes (2 hospitals in one country)	Open-label	Superiority	Controlled Cord Traction, Hands-off	Median blood loss primary, sPPH and PPH secondary	Drape-weight	204	199
Active Management trial (Gülmezoglu 2012[19])	RCT	Yes (8 countries worldwide)	Open-label	Non-inferiority	Simplified Package, Full Package	sPPH primary, PPH secondary	Drape-weight	24390	23242
CHAMPION trial (Widmer 2018[18])	RCT	Yes (10 countries worldwide)	Double-blind	Non-inferiority	Carbetocin, Oxytocin	sPPH primary, PPH secondary	Drape-weight	29645	29470
Bamberg et al study (Bamberg 2016 [21])	Observational	No	-	-	-	sPPH, PPH	Drape-Volume	1019	809

* Trial sample size is the total number of women enrolled in the trial; Effective sample size is the number of women with blood loss measure available.
