# Supplementary material for: Distribution of postpartum blood loss: modeling, estimation and application to clinical trials
Source: Reprod Health. 2018 Dec 4;15:199. doi: 10.1186/s12978-018-0641-1 (PMC6280488; doi:10.1186/s12978-018-0641-1)
Supplement: Supplementary file 2 — Table A2. Goodness of fit statistics for the three-parameter lognormal and other distributions, by treatment (a: Misoprostol trial; b: Active Management trial; d: Althabe et al. trial), or aggregated treatments (c: CHAMPION trial). (PDF 134 kb) [file 12978_2018_641_MOESM2_ESM.pdf]

Table A2. Goodness of fit statistics for the three-parameter lognormal and other distributions, by treatment for the Misoprostol trial, the Active Management trial and the Althabe trial, and for aggregated treatments for the CHAMPION trial

**Trial**	**Treatment**	**Distribution**	**AIC***	**-2Loglikelihood**	**BIC***
Misoprostol	Misoprostol	Three-parameter Lognormal	122626.20	122620.19	122647.58
		Log Generalized Gamma	122639.50	122633.50	122660.88
		Lognormal	122668.73	122664.73	122682.98
		Loglogistic	122747.84	122743.84	122762.10
		Weibull	123996.77	123992.77	124011.03
		LEV	124454.15	124450.15	124468.41
	Oxytocin	Three-parameter Lognormal	120155.96	120149.96	120177.35
		Log Generalized Gamma	120361.89	120355.89	120383.28
		LEV	122058.99	122054.99	122073.25
Active Management	Simplified Package	Three-parameter Lognormal	153067.61	153061.61	153089.69
		Log Generalized Gamma	153203.14	153197.14	153225.22
		LEV	154419.72	154415.71	154434.44
	Full Package	Three-parameter Lognormal	152454.39	152448.39	152476.47
		Log Generalized Gamma	152623.15	152617.15	152645.23
		LEV	154008.31	154004.31	154023.03
CHAMPION	Aggregated	Three-parameter Lognormal	263897.57	263891.57	263921.36
		Log Generalized Gamma	264203.15	264197.15	264226.94
		LEV	269155.78	269151.78	269171.64
Althabe et al	Hands Off	Three-parameter Lognormal	1312.4056	1306.1503	1319.9052
		Lognormal	1312.7782	1308.6518	1317.8218
		Loglogistic	1314.8331	1310.7068	1319.8767
		LEV	1326.3266	1322.2003	1331.3702
	CCT	Three-parameter Lognormal	1332.0212	1325.7738	1339.6192
		Lognormal	1335.2346	1331.1121	1340.3424
		Loglogistic	1336.5424	1332.4199	1341.6502
		LEV	1354.9820	1350.8595	1360.0898

* AIC: Akaike Information Criterion; BIC: Baysian Information Criterion
