# Supplementary material for: Distribution of postpartum blood loss: modeling, estimation and application to clinical trials
Source: Reprod Health. 2018 Dec 4;15:199. doi: 10.1186/s12978-018-0641-1 (PMC6280488; doi:10.1186/s12978-018-0641-1)
Supplement: Supplementary file 3 — Table A3. Quantiles for the fitted three-parameter lognormal and empirical distributions, with 95% CI, by treatment (a: Misoprostol trial; b: Active Management trial; d: Althabe et al. trial), or aggregated treatments (c: CHAMPION trial). (PDF 145 kb) [file 12978_2018_641_MOESM3_ESM.pdf]

Table A6. Quantiles for the fitted three-parameter lognormal and empirical distributions with 95% CI, by treatment for the Misoprostol trial, the Active Management trial and the Althabe et al trial, and for aggregated treatments for the CHAMPION trial

| | | | Three-parameter lognormal distribution | | | Empirical distribution | | |
|---|---|---|---|---|---|---|---|---|
| **Trial** | **Treatment** | **%** | **Quantile** | **95% CI** | | **Quantile** | **95% CI** | |
| | | | | **Lower limit** | **Upper limit** | | **Lower limit** | **Upper limit** |
| **Misoprostol** | **Misoprostol** | 1 | 42.1 | 40.2 | 43.8 | 50 | 44 | 50 |
| | | 5 | 73.6 | 71.7 | 75.5 | 99 | 80 | 100 |
| | | 10 | 97.8 | 95.7 | 99.9 | 100 | 100 | 100 |
| | | 25 | 155.2 | 152.5 | 157.9 | 150 | 150 | 160 |
| | | 50 | 255.9 | 252.0 | 259.9 | 250 | 244 | 250 |
| | | 75 | 418.5 | 411.7 | 425.4 | 400 | 400 | 403 |
| | | 90 | 648.7 | 635.9 | 662.1 | 680.6 | 650 | 700 |
| | | 95 | 842.2 | 822.9 | 862.5 | 900 | 870 | 900 |
| | | 99 | 1372.1 | 1330.6 | 1415.8 | 1400 | 1360 | 1500 |
| | **Oxytocin** | 1 | 34.9 | 33.2 | 36.5 | 39 | 33 | 41 |
| | | 5 | 63.2 | 61.5 | 64.9 | 55 | 50 | 70 |
| | | 10 | 84.7 | 82.9 | 86.6 | 100 | 100 | 100 |
| | | 25 | 135.3 | 132.9 | 137.6 | 140 | 140 | 145 |
| | | 50 | 222.9 | 219.5 | 226.4 | 200 | 200 | 200 |
| | | 75 | 362.8 | 357.0 | 368.7 | 350 | 350 | 360 |
| | | 90 | 558.6 | 547.8 | 570.0 | 560 | 539 | 600 |
| | | 95 | 721.9 | 705.6 | 739.0 | 800 | 739 | 800 |
| | | 99 | 1164.5 | 1129.7 | 1201.2 | 1328.8 | 1250 | 1400 |
| **Active Management** | **Simplified Package** | 1 | 17.6 | 15.5 | 19.6 | 9.4 | 9.4 | 14.2 |
| | | 5 | 52.3 | 50.4 | 54.1 | 51.9 | 47.2 | 53.8 |
| | | 10 | 77.8 | 75.8 | 79.7 | 81.1 | 78.3 | 84.9 |
| | | 25 | 135.7 | 133.3 | 138.2 | 139.6 | 135.8 | 141.5 |
| | | 50 | 232.2 | 228.8 | 235.5 | 234.0 | 229.2 | 235.8 |
| | | 75 | 379.4 | 374.0 | 384.8 | 367.9 | 361.3 | 375.5 |
| | | 90 | 577.1 | 567.4 | 587.2 | 576.4 | 566.0 | 584.9 |
| | | 95 | 736.9 | 722.4 | 752.0 | 753.8 | 729.2 | 768.9 |
| | | 99 | 1155.1 | 1124.6 | 1187.4 | 1244.5 | 1185.8 | 1324.5 |
| | **Full Package** | 1 | 14.8 | 12.8 | 16.7 | 9.4 | 9.4 | 9.4 |
| | | 5 | 47.2 | 45.4 | 48.9 | 47.2 | 47.2 | 47.2 |
| | | 10 | 71.3 | 69.5 | 73.1 | 75.5 | 72.6 | 75.5 |
| | | 25 | 126.6 | 124.3 | 129.0 | 129.2 | 125.5 | 132.1 |
| | | 50 | 219.9 | 216.7 | 223.2 | 217.9 | 215.1 | 224.5 |
| | | 75 | 364.4 | 359.1 | 369.8 | 355.7 | 349.1 | 361.3 |
| | | 90 | 561.0 | 551.3 | 571.1 | 566.0 | 549.1 | 575.5 |
| | | 95 | 721.6 | 707.0 | 736.8 | 726.4 | 709.4 | 749.1 |
| | | 99 | 1146.4 | 1115.3 | 1179.4 | 1244.5 | 1198.1 | 1329.2 |

| Trial | Treatment | % | Three-parameter lognormal distribution | | | Empirical distribution | | |
|---|---|---|---|---|---|---|---|---|
| | | | Quantile | 95% CI | | Quantile | 95% CI | |
| | | | | Lower limit | Upper limit | | Lower limit | Upper limit |
| **CHAMPION** | **Aggregated** | 1 | 9.6 | 8.8 | 10.5 | 1.9 | 0.9 | 2.8 |
| | | 5 | 30.9 | 30.1 | 31.7 | 21.7 | 19.8 | 22.6 |
| | | 10 | 47.9 | 47.0 | 48.7 | 36.8 | 35.8 | 37.7 |
| | | 25 | 89.7 | 88.5 | 90.9 | 81.1 | 79.2 | 83.0 |
| | | 50 | 166.7 | 164.9 | 168.6 | 161.3 | 158.5 | 163.2 |
| | | 75 | 297.9 | 294.6 | 301.2 | 294.3 | 289.6 | 299.1 |
| | | 90 | 492.9 | 486.3 | 499.7 | 479.2 | 470.8 | 487.7 |
| | | 95 | 663.0 | 652.5 | 673.9 | 650.9 | 637.7 | 670.8 |
| | | 99 | 1148.5 | 1123.7 | 1174.4 | 1168.7 | 1132.1 | 1222.6 |
| **Althabe et al** | **Hands Off** | 1 | 104.6 | 87.2 | 121.4 | 101.5 | 101.5 | 1624.3 |
| | | 5 | 135.8 | 116.4 | 153.7 | 123.9 | 101.5 | 159.8 |
| | | 10 | 160.0 | 139.2 | 180.5 | 159.2 | 124.1 | 189.9 |
| | | 25 | 217.4 | 193.6 | 245.8 | 228.9 | 186.1 | 253.8 |
| | | 50 | 318.7 | 286.6 | 361.7 | 310.2 | 274.5 | 377.9 |
| | | 75 | 483.3 | 422.9 | 562.3 | 487.4 | 423.0 | 564.0 |
| | | 90 | 717.9 | 609.2 | 888.9 | 756.1 | 562.1 | 1026.5 |
| | | 95 | 915.9 | 753.3 | 1194.8 | 1027.0 | 753.9 | 1624.3 |
| | | 99 | 1460.6 | 1109.6 | 2139.3 | 1624.3 | 101.5 | 1624.3 |
| | **CCT** | 1 | 96.4 | 73.8 | 108.9 | 95.9 | 95.9 | 1863.1 |
| | | 5 | 120.6 | 103.4 | 134.7 | 109.6 | 95.9 | 139.1 |
| | | 10 | 140.0 | 124.6 | 156.8 | 139.9 | 107.2 | 157.9 |
| | | 25 | 188.1 | 166.4 | 212.3 | 191.8 | 150.4 | 218.1 |
| | | 50 | 277.5 | 244.1 | 315.9 | 282.0 | 242.5 | 319.6 |
| | | 75 | 430.8 | 374.4 | 505.5 | 423.0 | 372.2 | 515.1 |
| | | 90 | 660.3 | 553.8 | 829.6 | 720.8 | 503.8 | 827.2 |
| | | 95 | 861.4 | 697.9 | 1144.0 | 824.4 | 723.8 | 1863.1 |
| | | 99 | 1439.1 | 1070.5 | 2158.1 | 1853.7 | 95.9 | 1863.1 |
